# Health Behaviors and Quality of Life of Cancer Survivors in Massachusetts, 2006: Data Use for Comprehensive Cancer Control

**Published:** 2009-12-15

**Authors:** Temeika L. Fairley, Helen Hawk, Snaltze Pierre

**Affiliations:** Division of Cancer Prevention and Control, Centers for Disease Control and Prevention; Massachusetts Department of Public Health, Boston, Massachusetts; Massachusetts Department of Public Health, Boston, Massachusetts

## Abstract

**Introduction:**

Nearly 12 million cancer survivors are living in the United States. Few state-based studies have examined the health status and health-related quality of life (HRQOL) of this growing population. The objective of this study was to use Massachusetts Behavioral Risk Factor Surveillance System (BRFSS) data to describe cancer survivors' demographics, health behaviors, quality of life, use of preventive care services, and influenza vaccination rates.

**Methods:**

The demographic characteristics of cancer survivors and respondents without cancer were estimated on the basis of responses to questions in the 2006 Massachusetts BRFSS. We used multivariate logistic regression to compare health behaviors, comorbidities, quality of life, and cancer screening and influenza vaccination rates for cancer survivors compared with respondents who did not have cancer.

**Results:**

Cancer survivors and respondents who did not have cancer had similar rates of health behavioral risk factors including smoking, obesity, and physical activity. Rates of chronic disease (eg, heart disease, asthma) and disability were higher among cancer survivors. Cancer survivors reported higher rates of influenza vaccination and breast, colorectal, and cervical cancer screening than did respondents who did not have cancer. Survivors' self-reported health status and HRQOL (physical and mental health) improved as length of survivorship increased.

**Conclusion:**

This state-based survey allowed Massachusetts to assess health-related issues for resident cancer survivors. These findings will help state-based public health planners develop interventions to address the long-term physical and psychosocial consequences of cancer diagnosis and treatment.

## Introduction

Nearly 12 million cancer survivors are living in the United States (1). Approximately 66% of cancer patients are expected to live at least 5 years after diagnosis (2). As use of cancer screening tests increases, cancer treatments improve, and the US population ages, we can expect the number of cancer survivors to increase (3). Concerns about the long-term physical, psychological, and economic effects of cancer treatment on cancer survivors and their families are being recognized and addressed by public, private, and nonprofit organizations (4). Increased recognition of the seriousness of these issues has contributed to the development of responsive public health strategies such as the publication of *A National Action Plan for Cancer Survivorship* (5) by the Centers for Disease Control and Prevention (CDC) and  *From Cancer Patient to Cancer Survivor: Lost in Transition* (6) by the Institute of Medicine.

CDC's National Comprehensive Cancer Control Program (NCCCP) funds states, tribes/tribal organizations, and selected US territories and associated Pacific Island jurisdictions to develop and implement local comprehensive cancer control plans (7). Most cancer plans include specific goals and objectives about survivorship (4). NCCCP programs need population-based data sources to assess the effectiveness of activities related to survivorship. Population-based data will allow state-specific analyses of the health behaviors of cancer survivors. To meet this need, CDC's Division of Cancer Prevention and Control is sponsoring cancer survivorship questions on the 2009 Behavioral Risk Factor Surveillance System (BRFSS) survey. Data from the 2006 Massachusetts BRFSS survey are used in the analysis presented in this article to describe the demographic characteristics, health status, health-related quality of life (HRQOL), and health behaviors of cancer survivors and respondents without cancer in Massachusetts.

## Methods

In the 2006 Massachusetts BRFSS, questions on cancer survivorship were asked of respondents aged 18 years or older. The initial question was "Have you ever been diagnosed with cancer?" and respondents could answer yes, no, or "don't know/not sure," or refuse to answer. If they answered yes, respondents were then asked, "What type of cancer were you diagnosed as having?" and were provided a list of choices (lung, colorectal, prostate, breast, cervical, ovarian or uterine, pancreatic, stomach or esophageal, liver/bile duct, urinary/bladder, non-Hodgkin lymphoma, leukemia, thyroid, oral cavity/pharynx, melanoma, or other [specify]). Respondents could select up to 3 cancers from this list. Respondents were also asked, "In what month and year were you last diagnosed with cancer?"

We created a variable indicating cancer prevalence from the questions on cancer that had been added by the state and from the core module question on prostate cancer. To estimate the prevalence among adults of a history of cancer, we calculated the weighted percentage of all types of cancers together and for main cancer types.

Cancer survivors were compared to respondents without cancer on the following demographic characteristics: age, race/ethnicity (categorized as white, non-Hispanic; black, non-Hispanic; and all other), sex, employment status (collapsed into employed for wages, out of work/unable to work, other, and retired), marital status (collapsed into currently married/living together and all other), and education level (collapsed into less than high school graduate and high school graduate or more).

We selected the following indicators of health behavior, HRQOL, and health status to assess cancer survivors and respondents without cancer: self-reported health status, disability status, leisure-time physical activity, smoking status, alcohol consumption, receipt of influenza vaccine in the past 12 months, and being up to date with age-appropriate cancer screenings. We collapsed self-reported health status to 2 levels (excellent, very good, and good; and fair and poor). HRQOL was measured by using the CDC healthy days measures (mean physically unhealthy days in the past 30 days and mean mentally unhealthy days in the past 30 days) (8). Disability status was grouped as having disability more than 1 year and other. Leisure-time physical activity or exercise during the past month (other than the respondent's regular job) and binge drinking (men having 5 or more drinks on 1 occasion, women having 4 or more drinks on 1 occasion) were grouped as yes and no. Smoking status was categorized as current (smoked at least 100 cigarettes in their lifetime and now smoke some days or every day), former (smoked at least 100 cigarettes in their lifetime and currently do not smoke), and never (have not smoked at least 100 cigarettes in their lifetime). Responses to the question about having received influenza vaccine in the past 12 months were grouped as yes and no. Cancer screening was assessed, including mammography in the past 2 years for women aged 40 years or older, Pap smears in the past 3 years for women aged 18 years or older, colorectal cancer screening among men and women aged 50 years or older (within the past 5 years for endoscopy) and prostate cancer screening within the past 2 years among men aged 50 years or older (9). We assessed changes in self-reported health status and HRQOL by time since diagnosis. The length of cancer survivorship was estimated as time since diagnosis to the date of completing the survey.

Data were weighted by using SAS version 9.1.3 (SAS Institute, Inc, Cary, North Carolina) to account for the complex sampling design. We used multivariate logistic regression models controlling for sex and age to assess the association between cancer and variables of interest (obesity, tobacco use, physical inactivity, screening use, vaccination use). Cancer survivors were compared to respondents without cancer as a reference group. Significance was set at *P* < .05.

## Results

Overall, 8,091 people completed the Massachusetts BRFSS in 2006. Of these respondents, nearly 10% (n = 780) reported having ever been diagnosed with cancer. We excluded respondents with unknown age at the time interview or unknown date of diagnosis, respondents who reported "don't know/not sure" or refused to answer the question, respondents with nonmelanoma skin cancers, and those with multiple cancers. The resulting sample included 231 men and 485 women. More than half of the cancer survivors reported being diagnosed 6 or more years ago (Table 1). Compared with respondents without cancer, more cancer survivors were older, female, and non-Hispanic white. Fewer survivors were employed for wages or insured by private insurance companies. We found no significant differences by education level or marital status (Table 1).

Breast, cervical/ovarian/uterine, colorectal, and thyroid cancers and melanoma were the most frequently reported cancers among women (Table 2). Among men, prostate, colorectal, and bladder cancers; melanoma; and non-Hodgkin lymphoma were the most frequently reported. Cancer sites were reported as "unknown" by 9% of men and 3% of women who reported they had a history of cancer.

In the multivariate regression analysis, cancer survivors had similar behavioral risks, such as for smoking, drinking, and being obese, compared with respondents without cancer, but were less engaged in leisure-time physical activity (Table 3). Rates of heart disease, asthma, and disability were higher in survivors than in respondents without cancer. Cancer survivors were also significantly more likely to receive age-appropriate cancer screenings (except for prostate cancer screening) and influenza vaccination than respondents without cancer.

Survivors' self-reported health status and HRQOL (physical and mental health) improved significantly as length of survivorship increased (Figure). For example, the odds ratio for life satisfaction increased from 0.7 at 5 years or fewer since diagnosis to 1.3 at more than 10 years since diagnosis.

**Figure 1:**
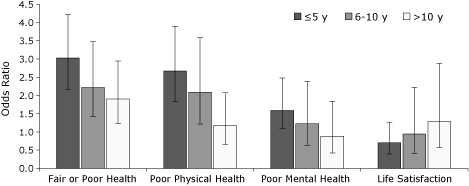
Health status and quality of life among cancer survivors, by the time since diagnosis, Massachusetts Behavioral Risk Factor Surveillance System, 2006. The reference group is respondents with no cancer diagnosed. Rates are adjusted for age and sex. I-bars represent 95% confidence intervals.

**Table:** 

**Health Status**	**Time Since Cancer Diagnosis**					
	**≤5 y**		**6-10 y**		**>10 y**	
	**OR**	**95% CI**	**OR**	**95% CI**	**OR**	**95% CI**
Fair or poor health	3.0	(2.2-4.2)	2.2	(1.4-3.5)	1.9	(1.2-2.9)
Poor physical health	2.7	(1.8-3.9)	2.1	(1.2-3.6)	1.2	(0.7-2.1)
Poor mental health	1.6	(1.1-2.5)	1.2	(0.6-2.4)	0.9	(0.4-1.8)
Life satisfaction	0.7	(0.4-1.3)	0.9	(0.4-2.2)	1.3	(0.6-2.9)

## Discussion

Historically, comprehensive cancer control programs have relied on cancer incidence, mortality, and local survey data to describe cancer in relation to cancer survivorship. The use of self-reported cancer prevalence data for cancer control at the state level is rare because few programs have the capacity to collect these data. We found that most cancer survivors were aged 55 years or older, regardless of sex. This pattern was similar to that documented in the Massachusetts Cancer Registry (MCR) (Helen Hawk, PhD, written communication, December 18, 2008). The distribution of self-reported cancers was also similar to that documented in the MCR (Helen Hawk, PhD, written communication, December 18, 2008). However, we observed variation in rankings of these cancers between these 2 systems. Lung cancers were more frequently documented by the MCR than the BRFSS. Lung cancer patients may be institutionalized or too sick to participate in the BRFSS telephone interviews. Their absence from survey data should be investigated through studies of data from, for example, caregivers and hospitals.

Time since diagnosis affected self-reported health status and quality of life among cancer survivors. These findings are similar to those of a previous study that assessed variation in HRQOL by time since diagnosis (10). However, they differ from findings from several other studies (11-13) that described health-related behaviors, HRQOL, and access to care among cancer survivors, indicating that cancer survivors have a lower quality of life than respondents without cancer.

Cancer survivors are also at increased risk of developing second cancers because of risk factors that led to the first cancer or as a consequence of therapy (14). These risk factors have also been linked to treatment complications, reduced quality of life, and mortality among cancer survivors (3,6,8,15). Since smoking cessation and increased exercise are associated with lower levels of cancer recurrence (8,15), appropriate activities aimed at improving or modifying these health behaviors may improve the health of Massachusetts cancer survivors.

Cancer survivors may also have increased risk for chronic conditions such as heart disease (3), diabetes (16), obesity-related asthma (17), and disability (18). Little is known about the effect of comorbid health conditions on diagnosis, treatment, subsequent health, or quality of life of cancer survivors; thus, further investigation into these relationships is warranted.

Cancer survivors in Massachusetts were more likely than respondents without cancer to receive age-appropriate screening for colorectal and cervical cancers, a finding similar to one in a previous study (19). However, the respondents did not differ significantly in receipt of screening for prostate and breast cancers, which differs from the findings in a study reporting that survivors were more likely to receive breast and prostate cancer screening than other respondents (19). Although screening guidelines recommend that young survivors receive screening at earlier ages (20), the small sample size prevented us from examining screening use in this population. Future analyses, which will include multiple years of data, may allow us to assess screening behavior in younger respondents.

Influenza vaccination is recommended for people with chronic diseases (21). Cancer survivors are at increased risk of developing complications from influenza (22). Therefore we examined vaccination use among Massachusetts survivors. We found that cancer survivors were significantly more likely than respondents without cancer to report receipt of the influenza vaccine. Although we did not assess the effect of age on vaccine use, prior studies noted that even in age-appropriate adults (23) only 59.2% of cancer survivors reported receiving an influenza vaccination. These rates may be appropriate, however, depending on the time since diagnosis and whether cancer patients are being actively treated for cancer (23).

Our findings are subject to several limitations. First, the survey may not be representative of people who do not have a land-line telephone, which is required for participation in the BRFSS survey (24). Second, BRFSS data are self-reported and subject to recall bias, which could lead to inaccurate estimates of cancer prevalence (25). Third, because our findings are limited to noninstitutionalized US citizens, cancer survivors who may have advanced disease and are living in nursing homes, long-term–care facilities, or hospice are not included in our study. Fourth, because this survey does not collect information from people younger than 18 years; thus, we are unable to describe the health behaviors of this population. Fifth, low cooperation for the Massachusetts BRFSS survey may also limit the generalizability of our study findings to all cancer survivors living in Massachusetts. Although studies have concluded that the national survey findings are reliable and valid (26), the reliability and validity of state-level data have not been directly assessed. To accurately do so, state-level BRFSS prevalence estimates must be compared with prevalence estimates from state cancer registries. Sixth, we also lacked information about cancer stage at diagnosis and whether the cancer diagnosis led to the development of other chronic conditions (eg, heart disease, diabetes, asthma) or vice versa. Also, the number and intensity of HRQOL issues vary with the type of cancer (27). Finally, the experience of cancer survivors in Massachusetts may differ from that of others in the United States because more than 95% of Massachusetts residents have health insurance (28). Increased access to health care as a result of health care reform initiatives may affect the health behaviors, health status, and overall survivorship of people with cancer. Studies are needed to assess the effect of increased health care access on the health behaviors of cancer survivors.

State-level population-based data on the health and care of cancer survivors may be used by cancer control programs to tailor programs that meet the needs of cancer survivors. For example, the Massachusetts Comprehensive Cancer Prevention and Control Program (MCCPCP) and the Massachusetts Comprehensive Cancer Control Coalition's Survivorship Workgroup used their BRFSS data to help address potential challenges in the provision of health care and preventive services for cancer survivors (eg, treatment of chronic disease, risk factor education). The MCCPCP has continued to support the collection of BRFSS data for cancer survivors. The additional data may be used to identify the needs of Massachusetts cancer survivors in certain subpopulations (eg, racial/ethnic minority groups) or with certain cancer types (eg, breast, colorectal, melanoma). Such information will help us to develop interventions to improve the quality of care and quality of life of cancer survivors.

## Figures and Tables

**Table 1:** Demographic Characteristics^a^ of Cancer Survivors and Respondents Without Cancer, Massachusetts Behavioral Risk Factor Surveillance System, 2006

**Characteristic**	Cancer Survivors, % (95% CI)(N = 716)	Respondents Without Cancer, % (95% CI)(N = 7,375)
**Years since diagnosis**		
≤5	48.2 (43.5-52.9)	NA
6-10	24.6 (20.5-28.7)	NA
>10	27.2 (23.1-31.3)	NA
**Age, y**		
18-54	28.1 (23.4-32.8)	72.8 (71.4-74.2)
55-64	22.7 (18.7-26.7)	12.8 (11.8-13.8)
≥65	49.3 (44.5-54.1)	14.4 (13.4-15.4)
**Sex, %**		
Male	37.9 (33.2-42.5)	47.6 (46.5-50.2)
Female	62.1 (57.5-66.8)	52.4 (49.8-53.5)
**Race/ethnicity, %**		
Non-Hispanic white	94.3 (91.5-95.7)	84.0 (82.0-84.8)
Non-Hispanic black	2.0 (1.1-3.7)	3.5 (2.9-4.0)
Other^b^	3.7 (2.4-5.7)	12.5 (11.8-14.4)
**Marital status, %**		
Married/living together	60.5 (56.0-65.1)	61.9 (59.7-63.5)
All other	39.5 (34.9-44.0)	38.1 (36.5-40.3)
**Education level, %**		
Less than high school graduate	7.1 (4.9-9.3)	6.6 (5.8-7.4)
At least high school graduate	92.9 (90.7-95.1)	93.4 (92.6-94.2)
**Employment, %**		
Employed for wages	36.6 (31.8-41.3)	64.3 (63.8-67.7)
Out of work/unable to work	9.4 (6.5-12.3)	4.2 (3.6-4.7)
Other	8.4 (5.7-11.1)	13.2 (10.4-16.4)
Retired	41.9 (37.3-46.5)	13.6 (12.1-14.1)
**Insurance access, %**		
Private	39.2 (34.3-44.0)	70.1 (68.4-71.8)
Government	5.1 (2.5-7.6)	7.0 (5.8-8.2)
Other	1.9 (0.6-3.3)	3.8 (3.0-4.6)
Medicare	53.8 (49.0-58.7)	19.1 (17.8-20.4)

AAbbreviations: CI, confidence interval; NA, not applicable.

a For some characteristics, the unknown/refused category has been suppressed. Therefore, percentages may not total 100. Percentages are weighted to the population distribution.

b Other includes people of Hispanic ethnicity, American Indians/Alaska Natives, Asians/Pacific Islanders, other, and unknown race.

**Table 2:** Percentage Distribution of Leading Self-Reported Cancers by Sex, Massachusetts Behavioral Risk Factor Surveillance System, 2006^a^

Cancer site	Men, % (N = 231)	Women, % (N = 485)
Bladder	3	3
Breast	NA	38
Cervix, ovary, uterus	NA	27
Colon, rectum	10	7
Leukemia	2	1
Liver/bile duct	3	<1
Lung	2	3
Melanoma	11	5
Non-Hodgkin lymphoma	6	3
Oral	2	<1
Pancreas	2	<1
Prostate	33	NA
Stomach/esophagus	1	<1
Thyroid	<1	4
Other^b^	14	6
Unknown^c^	9	3

Abbreviation: NA, not applicable.

a All percentages are weighted by using SAS version 9.1.3 (SAS Institute, Inc, Cary, North Carolina) to account for complex sampling design.

b Includes all cancers with frequencies less than 1%.

c Includes cancers reported by respondents who did not know the type of cancer they had.

**Table 3:** Risk Factors, Chronic Conditions, and Prevention Measures Among Cancer Survivors, Behavioral Risk Factor Surveillance System, Massachusetts, 2006

Characteristic	AOR^a^,^b^ (95% CI)
**Risk factors**	
Current smoker	1.2 (0.9-1.6)
Binge drinker	1.0 (0.7-1.5)
Obese	1.1 (0.9-1.5)
No leisure-time physical activity	1.3 (1.1-1.7)
**Chronic conditions**	
Heart disease	1.5 (1.1-2.0)
Disability	1.5 (1.2-2.0)
Current asthma	1.4 (1.1-1.9)
Diabetes	1.1 (0.8-1.5)
**Prevention measures**	
Colorectal cancer screening^c^	1.4 (1.1-1.8)
Prostate cancer screening^d^	1.4 (0.9-2.2)
Cervical cancer screening^e^	1.4 (1.1-2.0)
Mammography^f^	1.5 (1.0-2.3)
Influenza vaccination^g^	1.6 (1.2-2.0)

Abbreviations: AOR, adjusted odds ratio; CI, confidence interval.

a Reference group is respondents with no cancer diagnosed.

b Rates are adjusted for age and sex.

c Endoscopic examination within the previous 5 years or a fecal occult blood test within the previous year for adults aged 50 years or older.

d Prostate-specific antigen test within the previous 2 years for men aged 50 years or older.

e Papanicolaou test in the previous 3 years for women aged 18 years or older.

f Mammography in the previous 2 years for women aged 40 years or older.

g Receipt of influenza vaccine in the previous 12 months.
